# Rhus and Safflower Extracts as Potential Novel Food Antioxidant, Anticancer, and Antimicrobial Agents Using Nanotechnology

**DOI:** 10.3390/foods8040139

**Published:** 2019-04-23

**Authors:** Faten Y. Ibrahim, Ayman Y. EL-Khateeb, Azza H. Mohamed

**Affiliations:** 1 Food Industries Department, Faculty of Agriculture, Mansoura University, Mansoura 35511, Egypt; faeem@mans.edu.eg; 2 Agricultural Chemistry Department, Faculty of Agriculture, Mansoura University, Mansoura 35511, Egypt; aymanco@mans.edu.eg; 3 University of Florida, IFAS, Citrus Research & Education Center, 700 Experiment Station Road, Lake Alfred, FL 33850, USA

**Keywords:** green nanoparticles synthesis, transmission electron microscope, HEPG2, T47D, Caco-2, Gram-positive bacteria, Gram-negative bacteria

## Abstract

Green synthesis of metal nanoparticles using plant extracts offers a safe and attractive alternate to the chemical methods. The present work aims at preparing metal nanoparticles of rhus (*Rhus coriaria* L.) and safflower (*Carthamus tinctorius* L.) extracts using Fe^2+^, Cu^2+^, Zn^2+^, and Ag^+^ ions. The water extracts were prepared, and the total polyphenols and flavonoids contents were determined. The safflower extract contained the highest number of total polyphenols and total flavonoids (87.20 mg GAE/g and 36.32 mg QE/g), respectively. The synthesized nanoparticles were characterized using UV–Visible (UV-Vis) spectroscopy and Transmission Electron Microscope (TEM). The studied extracts and their nanoparticles were evaluated as an antioxidant, antimicrobial, and anticancer agents. The plant extracts and their nanoparticles showed significant antioxidant activity using (3-ethylbenzothiazoline-6-sulfonic acid (ABTS^•+^) and 2, 2-diphenyl-1-picrylhydrazyl (DPPH) assays. Safflower silver nanoparticles (AgNPs) were the most powerful antimicrobial agent compared to the other nanoparticles. The Sulforhodamine B (SRB) cytotoxic activity was evaluated against three cancer cell lines. The results revealed that CuNP safflower nanoparticles displayed the highest activity as anticancer agent with values (98.94% with T47D, 97.68% with HEPG2, and 89.33% against Caco-2). The data revealed that rhus and safflower extracts and their nanoparticles possess high potential activity as antimicrobial, antioxidant, and anticancer agents.

## 1. Introduction

The area of nanotechnology research is emerging as a cutting-edge technology with chemistry, physics, biology, material science, and medicine [1]. The ecofriendly synthesis of metal nanoparticles using plant extracts was previously established by several studies [2,3]. Natural antioxidants, such as phenolic compounds, flavonoids, and lignans, have established a high level of consideration due to their unique benefits. Plants rich in these classes of compounds are perfect sources of natural antioxidants. Rhus and safflower were used as flavoring spice, traditional medicine, and food preservation [4]. Today, a considerable number of reports have shown that the addition of rhus and safflower in food or water can have valuable effects on human and animal health [5]. Safflower has been used for a long time as a basis for dietary fat, food coloring, and Chinese medicines. Safflower compounds have antioxidant activity by scavenging of free radicals’ species, such as the superoxide anion (O_2_^−^) [6] and DPPH [7,8], indicating the significance of safflower as a provenance of antioxidants. Food components can be protected from the oxidation that results from Reactive Oxygen Species (ROS) by using active ingredients of rhus and safflower as alternative antioxidants [9]. Although Kinobeon A, component that had never been isolated naturally from plants, animals or microorganisms, was previously isolated only from safflower cell culture [10].

North American Indians usually apply rhus in the remediation of bacterial diseases such as syphilis, streaming, purge, and gangrene [11]. British Columbia screened more than 100 medicinal plants for antibiotic activity and found that crude methanolic extracts of rhus had both the largest inhibition zones in a disk test and the widest spectrum of activity [12]. The antimicrobial activity of the methanolic extract and isolated ingredients of rhus was also estimated by Saxena et al. [13] and the extract was active against eleven microorganisms tested. Also, rhus have antifungal activity against the nine fungi tested in research to screen 100 methanolic plant extracts for antifungal activity [12].

The goal of this work is to produce nanoparticles of rhus and safflower extracts through green synthetic pathways using Fe^3+^, Zn^2+^, Ag^+^, and Cu^2+^, metal ions. Also, the total polyphenol and flavonoid contents of rhus and safflower extracts and their synthesized nanoparticles were determined. The plant extracts and their corresponding nanoparticles were evaluated as antioxidant, antimicrobial and anticancer agents.

## 2. Materials and Methods

### 2.1. Materials

Rhus and safflower were purchased from the local market of Mansoura city in Egypt. The plants were air dried to a level of 9.5 ± 0.3% final moisture content after that were ground to a fine uniform powder using the Braun GmbH mill (model KSM2; type 4041, Kronberg, Germany). The ground shriveled plants were passed through a fine sieve (75–100 μm) to isolate only the fine powder. The chemicals and reagents were obtained from the appropriate sources: phenolic reagent of FOLIN-CIOCALTEU (Fluka, Biochemical Inc., bucharest, Romania), MB Gallic Acid (Biomedical Inc., Orange City, FL, USA), DPPH (Aldrich Chemistry, Kappelweg 1, 91625 Schnelldorf, Germany), Sodium Carbonate (El-Nasr Pharmaceutical Chemicals, Cairo, Egypt), AgNO_3_ (BASF Chemical Co., Cheadle, England), ZnSO_4_ (Andenex-Chemie, Hamburg Germany), CuSO_4_, and FeCl_3_ (Alpha Chemika, Panvel, Maharashta, India).

### 2.2. Preparation of Plant Extracts

Extraction of rhus and safflower was performed according to the method described by Deve et al. [14]. Briefly, 5 g fine powder of each plant material was separately extracted in 100 mL of deionized water on a horizontal water bath (Memmert WB14, Aldrich Chemistry, Schnelldorf, Germany) at 60 °C for 30 min. The water extracts were then filtered on Whatman No.1 filter paper (Whatman International Ltd., Kent, UK) using a Buchner funnel and the filtrates were adjusted to 100 mL in volumetric flasks with appropriate deionized water and stored at −18°C until further analysis.

### 2.3. Metal Nanoparticles Synthesis

Iron, zinc, silver, and, copper nanoparticle metals (FeNPs, ZnNPs, AgNPs, and CuNPs, respectively) were synthesized ecologically according to the method described by Pattanayak and Nayak [3] and slightly modified by El-Refai, Ghoniem, El-Khateeb, and Hassaan [2]. Twenty milliliters of 1 mmol aqueous solution of each metal salt (silver nitrate, zinc sulfate, copper sulfate and ferric chloride were prepared separately using deionized water and added to 20 mL of prepared plant extract. The resulting nanoparticles were synthesized in an equimolar ratio of plant extracts (1:1) to ionic solutions. The reaction mixture was stirred for extra two hours at 25 °C. The mixtures were irradiated by using a special UV lamp with a reduction factor property (Vilber Lourmat-6.LC, VILBER Smart Imaging, Marne-la-Vallée, France) and at a wavelength (λ = 254 nm) for 10 min using the same procedure described by Sharma et al. [15]

### 2.4. Instrumental Analysis for Metals Nanoparticles

The Ag^+^, Zn^2+^, Cu^2+^, and Fe^3+^ metals of the obtained nanoparticles were capped and stripped as reported by El-Shahaby, et al. [16] using the ATI Unicom UV–Vis spectrophotometer (Labomed inc., Calver City, CA, USA). Spectra of the reaction mixture were recorded at various time terms. The UV–Visible spectra of the resulting AgNPs, ZnNPs, CuNPs, and FeNPs were recorded in the 200 to 800 nm range using ATI Unicom UV–Vis. V 3.20 vision software. The analysis was carried out at room temperature using quartz cuvettes (optical path of 1 cm), and the white color was the corresponding aqueous extracts of rhus and safflower.

### 2.5. Transmission Electron Microscope (TEM) Measurement

Nanoparticle morphological properties such as surface, size, shape, and aggregation were confirmed by TEM (JEOL TEM-2100, Tokyo, Japan) according to El-Refai, Ghoniem, El-Khateeb, and Hassaan [2]. The TEM measurements and images were performed at the Electronic Microscope Unit, College of Agriculture, Mansoura University, Mansoura, Egypt.

### 2.6. Determination of Total Polyphenols Content

The total phenolic content in the aqueous extracts was determined in the plant extracts and their corresponding nanoparticles using the FOLIN-CIOCALTEU phenolic reagent modified method according to Yadav and Agarwala [17]. Aliquots of 0.1 mL of the solution were taken and mixed with exactly 2.8 mL of distilled water, 2.0 mL of 2% (*w/v*) sodium carbonate, and finally, 0.1 mL of FOLIN-CIOCALTEU reagent at 50% (*v/v*). The mixture was incubated for 30 min at room temperature and the resulting color absorbance was measured at 750 nm against distilled water as blank using a spectrophotometer. For the quantitative determination, a standard curve of gallic acid (0–200 mg L^−1^) was prepared in the same way (Table S1). The total phenol contents were expressed in milligrams of gallic acid equivalent (GAE)/g dry weight.

### 2.7. Determination of Total Flavonoids Content

Total flavonoids were determined in the tested plant extracts and their nanoparticles using the colorimetric method according to Yadav and Agarwala [17] with some modifications. The plant materials (0.1 g) were homogenized in 1 mL of distilled water. The resulting solution (0.5 mL) was mixed with 1.5 mL of 95% ethyl alcohol, 0.1 mL of 10% aluminum chloride (AlCl_3_), 0.1 mL of potassium acetate 1M, and 2.8 mL of distilled water. After incubation at room temperature for 40 min, the absorbance of the reaction mixture was measured at 420 nm against distilled water as white, using a spectrophotometer. The total flavonoid content was determined and expressed in milligrams equivalent quercetin (QE) g^−1^ dry weight using Quercetin (0–50 mg L^−1^) as the standard curve (Table S2).

### 2.8. Radical Scavenging Activity (RSA) Using DPPH Assay

The entrapment capacity of DPPH of each sample of the plant extracts and their produced nanoparticles was determined according to Li et al. [18]. The trapping capacity of the DPPH radicals was calculated according to the following equation.

$$\%\;\mathrm{Scavenging}\;\mathrm{activity}\;\left(\%\;\mathrm{RSA}\;\right)=\frac{\left(A_{0}\;“\mathrm{control}”\right)-\left(A\;\mathrm{sample}\right)}{A_{0}\;“\mathrm{control}”}\;\times \;100$$

where *A*_0_ is the absorbance of the control and *A* is the absorbance of the sample. Each sample was analyzed in triplicate.

### 2.9. Radical Scavenging Activity against ABTS^•+^

The trapping capacity of each sample of tested extracts and their corresponding nanoparticles was determined according to the method of Christodouleas et al. [19]. An appropriate amount of the ABTS^•+^ diammonium and the potassium persulfate salts were diluted to a final concentration of 7.00 and 2.45 mM, respectively. The solution was kept in the dark for 12 to 16 h for the formation of ABTS^•+^ radicals. Then, the solution of the ABTS^•+^ radical was diluted with ethanol to adjust the absorbance of the solution to 1.0. To determine the radical scavenging activity of the solutions (plant extract and their nanoparticles) against ABTS^•+^, the dilution step was required before measurements. Two milliliters of ABTS^•+^ alcoholic solution was added to 0.5 mL of each sample. The absorbance was then measured after 15 min at 734 nm. At least five measurements were performed for each sample and the% RSA was also calculated using the previous equation.

### 2.10. Microbial Susceptibility Testing

The antimicrobial activity test was performed in the biotechnology and genetics unit at Mansoura University. Using bacteria, such as *Proteus vulgaris* (PV); *Staphylococcus aureus*, *Erwinia carotovora* (EC), *Bacillus subtilis*, *Klebsiella pneumonia* (BS), and *Candida albicans* (CA), inoculums containing 10^6^ bacterial and fungal cells or 10^8^ yeast cells mL^−1^ were extended on nutrient agar, Czapek Dox agar, and Sabouraud agar, respectively. The bacterial strains used in this study were provided from stock culture at the molecular biology Department where the test was done. The experiment was designed to use paper disks of Whatman No. 1, and 6 mm diameter filter paper discs were sterilized and used with an infusion. The disks were installed on the surface of the gel plates seeded with the tested organisms. Plates were incubated at 37 °C for bacteria and at 30 °C for yeast. Diameters of the inhibition zone (mm) were measured after 24 h for bacteria and 48 h for yeast [16].

### 2.11. Potential Sulforhodamine B (SRB) Cell Cytotoxicity Assay

The cytotoxic potential of the extracts and their nanoparticles was achieved using the procedure described by Gaidhani, et al. [20]. Cells were plated in 96-well multiple plates (10^4^ cells/well) for 24 h before treatment with the tested sample to facilitate attachment of the cell line to the plate wall. Each concentration of the tested samples (100, 250, and 500 μg mL^−1^) was transferred to the monolayer well cells and incubated with the sample for 48 h at 37 °C under a carbon dioxide (5%) atmosphere. After 48 h, cells were fixed, washed, and stained with sulforhodamine B staining [20]. The excess stain was washed with acetic acid and the stain attached was recovered with tris-EDTA buffer. The intensity of the color was recorded in an ELISA reader. The relationship between the treatment concentration in μg mL^−1^ and the surviving fraction is plotted to obtain the survival curve of each tumor cell line. The IC_50_ (the half maximal inhibitory concentration) was determined using the standard curve. IC_50_ is the concentration at which the curve passes the 50% inhibition level. It is commonly used as a measure of antagonist drug potency in pharmacological research. According to the FDA, IC_50_ represents the concentration of a drug (anticancer for example) that is required for 50% inhibition in vitro. The cancer cell lines source and the biological evaluation were provided by Oncology Department, National Cancer Institute Cairo University, Cairo, Egypt.

### 2.12. Statistical Analysis

The statistical analysis was done using the Co-Statistical Package [21]. The analysis of variance (ANOVA), split block, was performed to compare the tested samples and treatments. The significance of differences between the means was done using the Duncan multiple interval tests at *p* ≤ 0.05 [22].

## 3. Results and Discussion

The use of plant extracts as natural preservatives in the field of food industries has significantly increased recently. Also, plant extracts have been used to inhibit microbial growth and lipid oxidation. Aqueous extracts of rhus and safflower have been reported as potent antioxidants and antibacterial against foodborne pathogenic bacteria [23]. Safflower and rhus extracts were prepared at a concentration of 35 μg mL^−1^, which made it possible to prepare the metal nanoparticles. Using plant extracts as safflower and rhus extracts to obtain the metal nanoparticles is considered a safe method to reduce the tested ions and to prevent their corresponding nanoparticles from aggregation [2]. This green method provides an alternative method to the artificial methods that depend on chemical components to form and stabilize the nanoparticles [2]. Also, antimicrobial activities and anticancer effects were performed using the plant’s aqueous extracts and their corresponding nanoparticles.

### 3.1. Nanoparticles Characteristics via UV-Vis Spectroscopy

**Figure 1:**
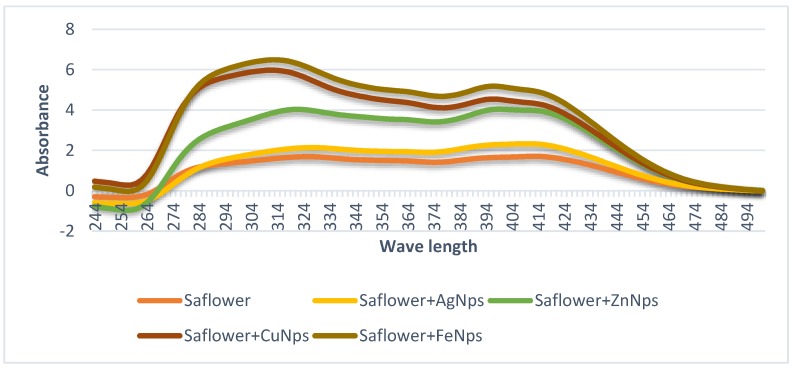
UV–Vis spectroscopic measurements of safflower and its prepared metal nanoparticles.

**Figure 2:**
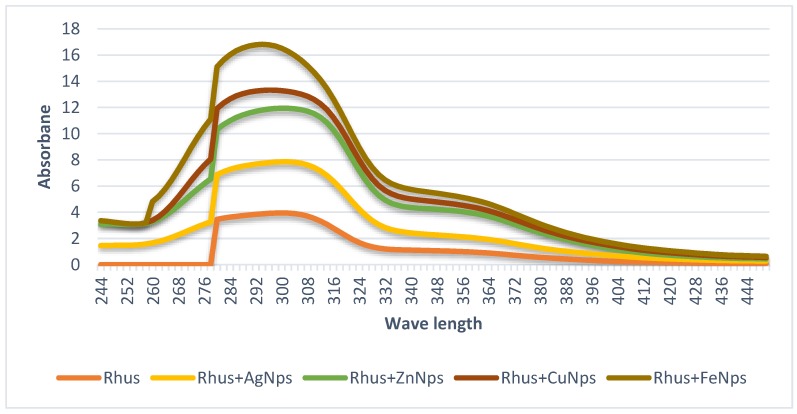
UV–Vis spectroscopic measurements of rhus and its prepared metal nanoparticles.

The synthesis of rhus nanoparticles (AgNP, ZnNP, CuNP, and FeNPs) in Figure 1 and safflower nanoparticles (AgNP, ZnNP, CuNP, and FeNPs) in Figure 2 was confirmed by measuring the UV–Vis spectrum of the reaction mixture. The stability of the produced nanoparticles was due to possible presence of the polyphenolic compounds [2]. The absorption peak at ~300 nm corresponds to the surface plasmon resonance characteristic of the resulted AgNPs, ZnNPs, CuNPs and FeNPs. El-Refai, Ghoniem, El-Khateeb, and Hassaan [2] stated that in the case where the same nanoparticle metal ions were prepared using garlic and ginger extract, the maximum absorption peak was at 280 nm. The nanoparticles obtained from rhus and safflower extracts were proven to be very stable, probably due to the polyphenolic compounds presented in the extracts that prevent agglomeration (Figure 1 and Figure 2). Rhus and safflower extracts are rich in flavonoids and phenolic compounds. Flavonoids play a key role in the process of reducing the synthesis of AgNPs, ZnNPs, CuNPs, and FeNPs [24,25]. Thus, the high levels of flavonoids and phenolic compounds in the rhus and safflower water extracts strongly confirmed the potential of rhus and safflower to bioreduce Ag^+^, Zn^2+^, Cu^2+^, and Fe^3+^ in AgNPs, ZnNPs, CuNPs, and FeNPs nanoparticles, respectively.

### 3.2. Nanoparticles Characteristic via Transmission Electron Microscope (TEM)

**Figure 3:**
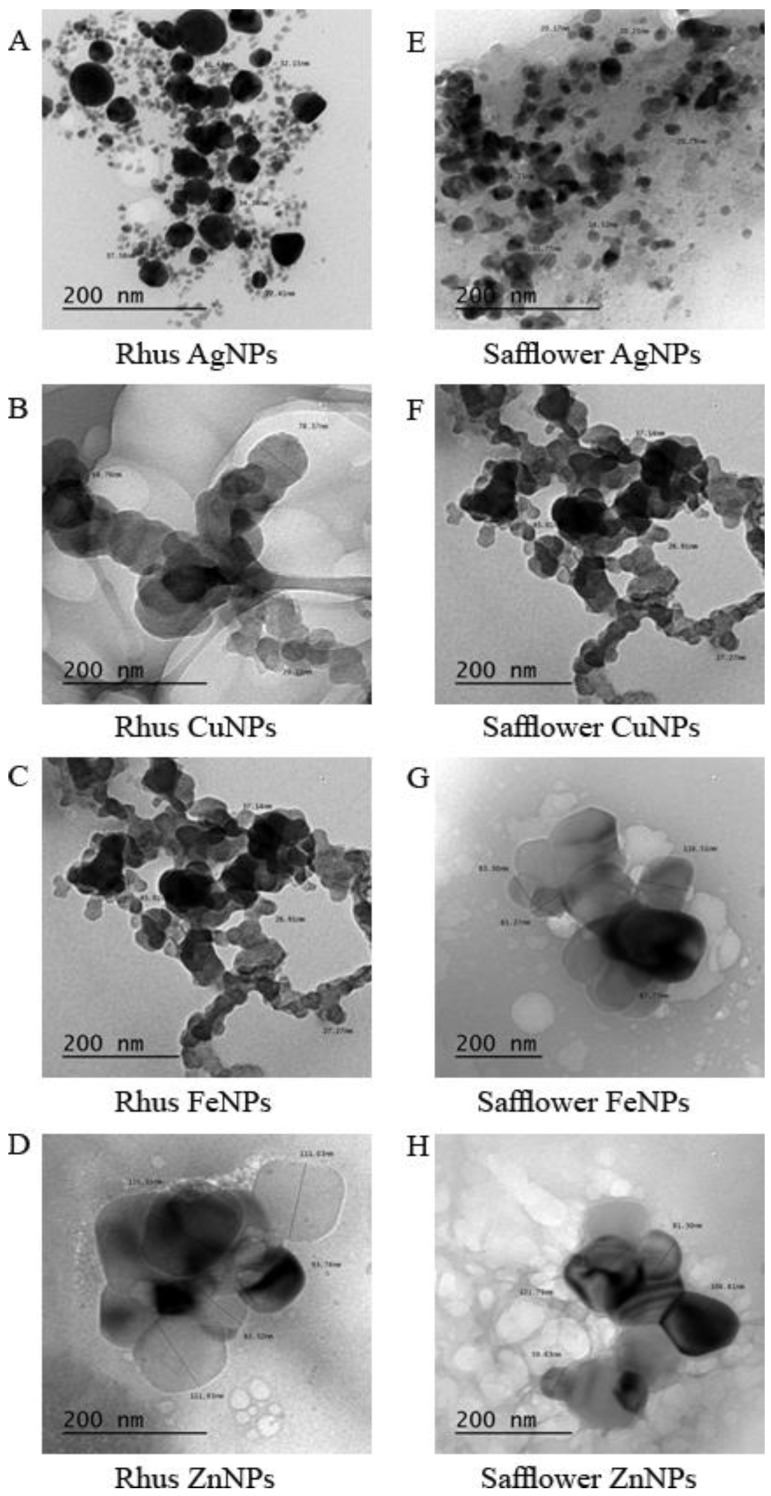
TEM micrographs characterization illustrating the size and morphology. AgNPs (**A**), CuNPs (**B**), FeNPs (**C**), and ZnNPs (**D**) obtained with rhus extracts and AgNPs (**E**), CuNPs (**F**), FeNPs (**G**), and ZnNPs (**H**) obtained with safflower extracts. Scale bar = 200 nm.

The nanoparticles of rhus and safflower extracts were prepared using Ag^+^, Cu^2+^, Fe^3+^, and Zn^2+^ metals. The nanoparticles were characterized by TEM measurements to confirm the formation of the nanoparticles and to characterize the form and aggregation of the synthesized nanoparticles [2]. TEM images were taken at 200 nm (Figure 3A–H). The size of AgNPs from rhus extract was between 22.41 and 37.58 nm (Figure 3A). In addition the size of the AgNPs of safflower was ~14.52–35.77 nm, with a spherical shape and a small number of tetragonal particles (Figure 3E). The synthesized nanoparticles of the AgNPs nanoparticles from rhus extract appeared to be more aggregated compared to the AgNPs particles of safflower (Figure 3A,E). The CuNPs particles from rhus showed size ranged from 29.15 to 78.37 nm (Figure 3B). These values are higher than those obtained from safflower CuNPs (26.91–45.01 nm) (Figure 3F). Safflower CuNPs also contains a larger number of small particles compared to rhus CuNPs and that might explain their high efficiency as anticancer.

The TEM measurements showed that the shape of rhus CuNPs nanoparticles was spherical and linear aggregated particles (Figure 3B). However the safflower CuNPs particles were formed in tetragonal with square aggregation shape of the particles (Figure 3F). The particles are found to be more aggregated in rhus CuNPs than safflower CuNPs with higher particles size. The particle size of rhus FeNPs ranged from 70.39 to 122.47 nm with the spherical and tetragonal shapes aggregated together (Figure 3C). On the other hand, the size of safflower FeNPs ranged from 67.77 to 116.51 nm with almost spherical, tetragonal, and pentagonal shapes (Figure 3G). The FeNPs nanoparticles of safflower has a high aggregation of particles in which the particles formed a cloud around the aggregation center. It was noticed that the particles appear more aggregated in the two plant extracts, with a larger size for rhus FeNPs. In conclusion, the nanoparticles for both extracts with zinc appear to be greater than those of other metals. The size of rhus ZnNPs particles ranged from 82.32 to 115.91 nm (Figure 3D), which higher than those of safflower ZnNPs particles (59.63–121.75 nm) (Figure 3H). It was proven that the obtained nanoparticles were aggregated into spherical and tetragonal forms for rhus ZnNPs and spherical, tetragonal, and linear forms in safflower ZnNPs. In general, the largest size of the synthesized particles is rhus FeNPs (122.47 nm) as shown in Figure 3C, and the smallest particles size is recorded for safflower AgNPs of (14.52 nm), as shown in Figure 3 E. Also, the Ag metal forms the smallest particle size with both extracts. The size of the synthesized nanoparticles was proven to be highly this is dependent on the nature of each metal (Figure 3A–H).

### 3.3. Determination of Total Polyphenolic and Flavonoid Contents

**Figure 4:**
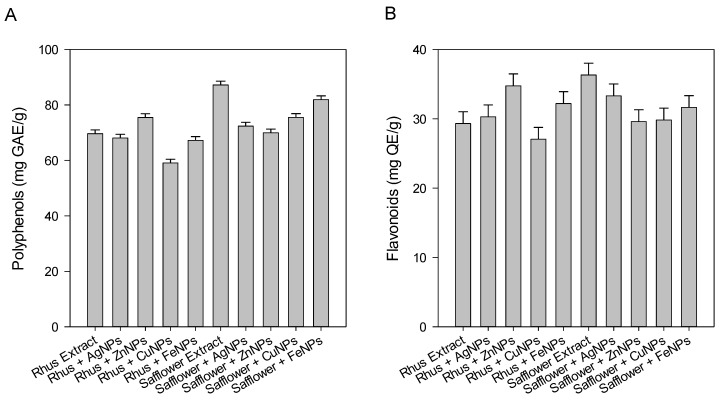
(**A**) Total polyphenolics (mg GAE g^−1^) and (**B**) total flavonoids (mg QE g^−1^) of rhus and safflower extracts and their nanoparticles.

In light of the consequences of rhus and safflower use in food recipes, alternative medicines, and the food industry, the chemical composition of rhus and safflower has long been explored and known as an abundant source of tannins, phenolic acids, anthocyanins, derivatives of gallic acid, flavonoids, glycosides, and organic acids [26]. Phenolic compounds have played an important role as antioxidant compounds and by preserving various foods from damage by inhibiting or killing harmful microorganisms. It has been noted that safflower extract and its FeNPs have the highest content of total polyphenols (Figure 4A). The total phenols and antioxidant activity of the rhus extract are much lower than those of the safflower extracts (Figure 4A).

Polyphenols include several classes of phenolic compounds that are secondary plant metabolites and are integral to human and animal diets. Safflower extract and its FeNP contain the highest number of total polyphenols 87.20 and 81.91 mg GAE g^−1^, respectively. Also, the data showed that the total phenols and antioxidant activity of the rhus extract are much lower than those of the safflower extract.

Flavonoids are an important group of phenolic compounds consisting mainly of flavones and anthocyanins. Phenolic compounds play an important role in the prevention of body cells and organs from damage by hydrogen peroxide, damage by lipid peroxides and the recovery or neutralization of free radicals [27]. The total flavonoid content in Figure 4B showed that the safflower extract contained the highest number of total flavonoids, while rhus had the lowest number. Figure 4B showed that the mean values were ranging between 36.32 and 29.32 mg GAE g^−1^ for the safflower extract and rhus extract, respectively.

### 3.4. Antioxidant Activities of ABTS^•+^ and DPPH

**Figure 5:**
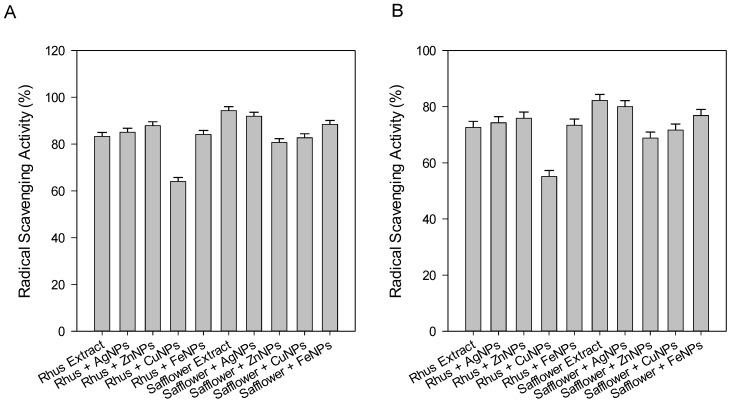
Comparison between the antioxidant results. (**A**) DPPH and (**B**) ABTS^•+^ of plant extracts and their synthesized nanoparticles at 35 µg/ mL.

The antioxidant activity of the extracts of rhus, safflower and their obtained nanoparticles were evaluated using the ABTS^•+^ and DPPH free radical tests. The results are presented in Figure 5A,B. The data in genaral indicated that all the rhus safflower extracts and their synthesized nanoparticles showed excellent antioxidant activity with a high percentage of radical scavenging activity in the range of 64.04% for ruhs CuNPs to 94.30% for sufflower extract using the DPPH assay and from 55.12% ruhs CuNPs to 82.17% for sufflower extract using the ABTS^•+^ assay (Figure 5A,B, respectively). El-Refai, Ghoniem, El-Khateeb, and Hassaan [2] reported that the ZnNps produced from ginger water extract was the best antioxidant agent with 77.31% and 63.90% radical scavenging activity using DPPH and ABTS^•+^ assays, respectively.

Based on the obtained results for antioxidants in the investigated samples, the high antioxidant capacity of the safflower extract and its AgNPs can be explained by its ability to trap the free radicals such as peroxide and alkoxyl radicals resulting from the effect of the compounds such as, phenolic and flavonoids [2]. Several previous researchers studied the antioxidants and concluded that (i) radicals produced from lipid peroxides [28,29] and interaction with other antioxidant compounds [30], (ii) antioxidant properties preventing oxidative attack on membrane lipids by sparing vitamin E or regenerating vitamin E as vitamin C does [31], (iii) inhibition of lipoxygenases [32,33] known to be stimulated by lipid peroxides and which may be involved in oxidative stress, and (iv) inhibition of cellular enzymes involved in signal transduction [32].

### 3.5. Antimicrobial Activity

The rhus and safflower extracts and their synthesized nanoparticles were tested for the antibacterial activity against the Gram-positive bacteria (*Bacillus subtilis* (BS) and *Staphylococcus aureus* (SA)) and the Gram-negative bacteria (*Erwinia carotovora* (EC), *Proteus vulgaris* (PV), and *Klebsiella pneumonia* (KP)). The plant extracts and their nanoparticles were tested against *Candida albicans* (CA) microorganism for their antifungal activity.

According to the results presented in Table 1, the highest antibacterial activity was in the case of the FeNPs nanoparticles from safflower against *S. aureus* with an inhibition zone of 16.8 mm, while the strongest antifungal agent was found in the safflower extract with an inhibition zone of 15.40 mm against CA strain. Moreover, rhus AgNPs and safflower AgNPs show high antibacterial activity against *E. carotovora* with inhibition zones of 10.70 mm and 10.50 mm, respectively. The AgNPs, CuNP, and FeNPs from safflower have the highest activity against Gram-negative bacteria (*P vulgaris* and *K. pneumonia*) as presented in (Table 1). Also, Cu^2+^ ion nanoparticles with safflower extract had the highest activity against *BS* (Gram-positive bacteria) with inhibition zone of 11.40 mm. Also, the nanoparticles for the safflower extract with Ag^+^ and Fe^3+^ had the highest activity to inhibit the growth of the *S. aureus* strains with inhibition zone of 14.30 mm and 16.80 mm, respectively. In general, most tested extracts of rhus and safflower and their synthesized nanoparticles show good inhibition against the tested bacterial and fungal strain (Table 1). In conclusion, the ability of the nanoparticles as antimicrobial and antifungal agents was determined by some factors such as the type of metal forming the nanoparticles, nature, and the physical properties of the nanoparticles. Also, it was found that the smaller particle size and the more aggregation of the particles, the faster the spread and the more efficient in killing the different microorganisms. These results agreed with the results reported by El-Refai, Ghoniem, El-Khateeb, and Hassaan [2].

### 3.6. Potential Sulforhodamine B (SRB) Cell Cytotoxicity Assay

The cytotoxic activities of rhus and safflower extracts, as well as their nanoparticles, were evaluated against known cancer cell lines, e.g., Caco-2, HEPG2, and T47D using the SRB assay (Table 2). The cytotoxicity values are measured at different concentrations (100, 250, and 500 μg mL^−1^) of extracts and their nanoparticles. The IC_50_ values expressed the 50% concentration of inhibitory concentration. The tested samples showed excellent to moderate activities with high percent inhibition and lower IC_50_ values. Increasing the concentration of the tested samples improves anticancer activity with a higher percentage inhibition. The data listed in Table 2 revealed that safflower copper nanoparticles are the highest anticancer agent against all the tested cell lines. On the other hand, the safflower extract and its nanoparticles have higher activity than the results obtained with rhus extract. AgNPs, ZnNPs, CuNPs, and FeNPs nanoparticles of safflower showed good anticancer activity against the Caco-2 cell line. Also, CuNPs and FeNPs from rhus and safflower extracts and nanoparticles with Ag, Zn, Cu, and Fe metal ions showed good activity against HEPG2 and T47D cell lines. Also, the nanoparticles prepared from each rhus extract and safflower have higher activity than the tested extract due to nature, particle size and aggregation that led to the improvement of the results. The smaller particle size can reach the tested cells with a higher concentration.

## 4. Conclusions

The extraction of rhus and safflower plants was used for the ecological preparation of nanoparticles with four metal ions: Zn^2+^, Cu^2+^, Ag^+^, and Fe^3+^. The evaluation of the antioxidants of the two extracts and their nanoparticles revealed that the safflower extract and its AgNPs had the highest trapping activity using the DPPH and ABTS^•+^ assays. The results of antioxidant activity using the DPPH assay revealed that all extracts have potent activities as antioxidants. Safflower and rhus extracts and their nanoparticles have been evaluated as antibacterial and antifungal agents. The results revealed that safflower extract and its AgNPs and CuNPs exhibited high potent activity against all the microorganisms tested due to the smaller particle size of the metal ions and the aggregation of the particles. The higher size of the zinc particles and metal iron ions reduces the antimicrobial activity of the extracts. Further evaluation of the extracts and their nanoparticles as anticancer agents has revealed that safflower extract and its AgNPs and CuNPs have potent activities against the Caco-2, HEPG2, and T47D cell lines. The higher the concentration of each extract, the more effective against different cell lines.

## Figures and Tables

**Table 1:** Antimicrobial activities of rhus and safflower extracts and their nanoparticles using agar disc diffusion technique at 35 µg mL^−1^ after 24 h.

Samples	Inhibition Zone (IZ) of Bacterial Strains (mm)					
	Gram-Negative Bacteria			Gram-Positive Bacteria		*Candida albicans*
	*EC* *	*PV* *	*KP* *	*BS* *	*SA* *	*CA* *
Rhus Extract	0.00 ± 0.00 ^b^**	12.80 ± 0.47 ^a^	7.00 ± 0.47 ^ab^	0.00 ± 0.00 ^b^	0.00 ± 0.00 ^b^	7.70 ± 0.47 ^ab^
Rhus + AgNPs	10.70 ± 0.47 ^ab^	8.40 ± 0.47 ^ab^	9.80 ± 0.47 ^ab^	9.50 ± 0.47 ^ab^	8.20 ± 0.47 ^ab^	11.40 ± 0.47 ^ab^
Rhus + ZnNPs	7.60 ± 0.47 ^ab^	7.20 ± 0.47 ^ab^	0.00 ± 0.00 ^b^	7.30 ± 0.47 ^ab^	7.70 ± 0.47 ^ab^	0.00 ± 0.00 ^b^
Rhus + CuNPs	8.70 ± 0.47 ^ab^	7.60 ± 0.47 ^ab^	7.50 ± 0.47 ^ab^	8.40 ± 0.47 ^ab^	0.00 ± 0.00 ^ab^	7.80 ± 0.47 ^ab^
Rhus + FeNPs	8.50 ± 0.47 ^ab^	0.00 ± 0.00 ^b^	8.20 ± 0.47 ^ab^	7.60 ± 0.47 ^ab^	8.50 ± 0.47 ^ab^	8.00 ± 0.47 ^ab^
Safflower Extract	9.40 ± 0.47 ^ab^	8.30 ± 0.47 ^ab^	7.50 ± 0.47 ^ab^	8.00 ± 0.47 ^ab^	8.20 ± 0.47 ^ab^	15.40 ± 0.47 ^a^
Safflower + AgNPs	10.50 ± 0.47 ^ab^	13.70 ± 0.47 ^a^	14.50 ± 0.47 ^a^	9.20 ± 0.47 ^ab^	14.30 ± 0.47 ^a^	9.20 ± 0.47 ^ab^
Safflower + ZnNPs	8.50 ± 0.47 ^ab^	0.00 ± 0.00 ^b^	7.70 ± 0.47 ^ab^	7.50 ± 0.47 ^ab^	7.60 ± 0.47 ^ab^	7.30 ± 0.47 ^ab^
Safflower + CuNPs	9.40 ± 0.47 ^ab^	12.50 ± 0.47 ^a^	13.00 ± 0.47 ^a^	11.40 ± 0.47 ^ab^	8.20 ± 0.47 ^ab^	9.40 ± 0.47 ^ab^
Safflower + FeNPs	7.50 ± 0.47 ^ab^	12.60 ± 0.47 ^a^	0.00 ± 0.00 ^b^	0.00 ± 0.00 ^b^	16.80 ± 0.47 ^a^	9.50 ± 0.47 ^ab^

* *Erwinia carotovora* (EC); *Proteus vulgaris* (PV); *Klebsiella pneumonia* (KP); *Bacillus subtilis* (BS); *Staphylococcus aureus* (SA); *Candida albicans* (CA). ** a,b statistical analysis as the mean comparison; Means with the same letter in a column are not significantly different at *p* ≤ 0.05.

**Table 2:** Anticancer activities of rhus and safflower extracts and their nanoparticles against Caco-2, HEPG2 and T47D cell lines.

Samples	Conc. (µg mL^−1^)	Cancer Cell Inhibition %			IC_50_ (µg mL^−1^)		
		Caco	HEPG2	T47D	Caco	HEPG2	T47D
Rhus Extract	100	6.28 ± 0.53 ^p^*	10.34 ± 0.87 ^s^	17.71 ± 1.23 ^p^	1405.67 ± 17.14 ^a^	1093.46 ± 11.37 ^a^	1055.24 ± 12.27 ^a^
Rhus Extract	250	12.24 ± 1.10 ^mn^	15.23 ± 0.74 ^r^	22.37 ± 0.92 ^o^			
Rhus Extract	500	19.72 ± 1.37 ^l^	26.98 ± 1.13 ^o^	30.72 ± 1.45 ^m^			
Rhus + AgNPs	100	7.97 ± 0.82 ^op^	23.77 ± 0.81 ^p^	24.22 ± 1.11 ^o^	1147.89 ± 13.75 ^b^	720.49 ± 9.89 ^b^	567.38 ± 9.14 ^e^
Rhus + AgNPs	250	11.17 ± 0.98 ^n^	32.32 ± 1.91 ^m^	29.33 ± 1.87 ^mn^			
Rhus + AgNPs	500	23.99 ± 1.47 ^k^	46.20 ± 2.50 ^i^	40.94 ± 2.12 ^l^			
Rhus + ZnNPs	100	9.04 ± 0.70 ^o^	30.18 ± 1.35 ^n^	27.47 ± 1.76 ^n^	1005.16 ± 10.52 ^e^	676.17 ± 8.21 ^c^	538.07 ± 10.78 ^d^
Rhus + ZnNPs	250	13.31 ± 0.96 ^mn^	36.59 ± 1.81 ^hl^	32.12 ± 1.30 ^m^			
Rhus + ZnNPs	500	27.19 ± 1.10 ^j^	48.34 ± 1.57 ^h^	43.26 ± 2.04 ^l^			
Rhus + CuNPs	100	18.65 ± 0.93 ^l^	66.49 ± 2.72 ^d^	47.44 ± 2.73 ^k^	721.91 ± 9.89 ^e^	169.52 ± 5.53 ^f^	3.58 ± 0.06 ^i^
Rhus + CuNPs	250	25.06 ± 1.23 ^jk^	71.83 ± 3.14 ^c^	52.55 ± 2.81 ^j^			
Rhus + CuNPs	500	38.94 ± 1.51 ^g^	91.06 ± 4.26 ^b^	66.94 ± 3.12 ^g^			
Rhus + FeNPs	100	5.83 ± 0.27 ^p^	35.52 ± 1.41 ^l^	31.19 ± 2.02 ^m^	926.05 ± 11.31 ^d^	443.45 ± 9.25 ^d^	342.33 ± 9.43 ^h^
Rhus + FeNPs	250	14.38 ± 0.81 ^m^	45.13 ± 1.84 ^ij^	40.94 ± 1.90 ^l^			
Rhus + FeNPs	500	27.19 ± 1.09 ^j^	59.02 ± 2.08 ^e^	52.55 ± 2.71 ^j^			
Safflower Extract	100	24.84 ± 1.19 ^jk^	14.38 ± 0.82 ^r^	44.34 ± 2.23 ^l^	615.77 ± 7.43 ^f^	202.29 ± 5.35 ^e^	707.75 ± 10.10 ^b^
Safflower Extract	250	32.27 ± 1.32 ^i^	22.92 ± 1.07 ^p^	52.88 ± 1.84 ^j^			
Safflower Extract	500	44.34 ± 1.56 ^e^	37.87 ± 0.91 ^k^	65.70 ± 3.16 ^g^			
Safflower + AgNPs	100	37.84 ± 1.22 ^gh^	30.40 ± 1.12 ^n^	58.22 ± 2.40 ^i^	282.09 ± 8.51 ^h^	18.24 ± 0.80 ^h^	359.21 ± 9.71 ^g^
Safflower + AgNPs	250	49.91 ± 1.74 ^d^	44.28 ± 1.64 ^j^	72.11 ± 3.21 ^f^			
Safflower + AgNPs	500	62.45 ± 1.85 ^c^	59.23 ± 2.04 ^e^	85.99 ± 3.80 ^d^			
Safflower + ZnNPs	100	41.09 ± 1.31 ^f^	33.60 ± 1.83 ^m^	62.49 ± 2.70 ^h^	261.52 ± 7.27 ^i^	7.18 ± 0.67 ^i^	386.49 ± 7.61 ^f^
Safflower + ZnNPs	250	49.91 ± 1.87 ^d^	45.35 ± 2.32 ^ij^	71.04 ± 3.01 ^f^			
Safflower + ZnNPs	500	62.45 ± 2.23 ^c^	54.96 ± 1.95 ^f^	90.26 ± 3.83 ^e^			
Safflower + CuNPs	100	65.23 ± 1.92 ^b^	67.78 ± 2.27 ^d^	83.85 ± 2.94 ^d^	3.12 ± 0.81 ^j^	1.11 ± 0.04 ^j^	1.42 ± 0.07 ^j^
Safflower + CuNPs	250	66.77 ± 2.34 ^b^	70.98 ± 2.70 ^c^	93.47 ± 3.52 ^b^			
Safflower + CuNPs	500	89.33 ± 3.28 ^a^	97.68 ± 3.44 ^a^	98.94 ± 4.03 ^a^			
Safflower + FeNPs	100	26.23 ± 1.27 ^jk^	19.72 ± 0.98 ^q^	48.61 ± 2.81 ^k^	498.32 ± 9.34 ^g^	99.61 ± 8.72 ^g^	487.45 ± 8.48 ^e^
Safflower + FeNPs	250	35.98 ± 1.81 ^h^	32.53 ± 1.23 ^m^	62.49 ± 0.87 ^h^			
Safflower + FeNPs	500	49.91 ± 2.09 ^d^	50.69 ± 1.80 ^g^	76.38 ± 0.82 ^e^			
LSD (*p* < 0.05)		1.95	1.40	2.62	3.40	1.51	9.42

* a-s The statistical analysis as the mean comparison; Means with the same letter in a column are not significantly different at *p* ≤ 0.05.

## Supplementary Materials

The following are available online at https://www.mdpi.com/2304-8158/8/4/139/s1, Table S1: Standard curve of gallic acid, Table S2: Standard Curve of Quercetin.

## References

[1] Devasenan S., Beevi N.H., Jayanthi S. (2016). Synthesis and characterization of silver nanoparticles by chemical reduction method and their antimicrobial activities. Int. J. ChemTech Res..

[2] El-Refai A.A., Ghoniem G.A., El-Khateeb A.Y., Hassaan M.M. (2018). Eco-friendly synthesis of metal nanoparticles using ginger and garlic extracts as biocompatible novel antioxidant and antimicrobial agents. J. Nanostructure Chem..

[3] Pattanayak M., Nayak P. (2013). Ecofriendly green synthesis of iron nanoparticles from various plants and spices extract. Int. J. Plant Anim. Environ. Sci..

[4] Abu-Reidah I.M., Jamous R.M., Ali-Shtayeh M.S. (2014). Phytochemistry, pharmacological properties and industrial applications of *Rhus coriaria* L. (sumac). Jordan J. Biol. Sci..

[5] Chakraborty A., Ferk F., Simić T., Brantner A., Dušinská M., Kundi M., Hoelzl C., Nersesyan A., Knasmüller S. (2009). DNA-protective effects of sumach (*Rhus coriaria* L.), a common spice: Results of human and animal studies. Mutat. Res. Fundam. Mol. Mech. Mutagenesis.

[6] Jia L.H., Liu Y., Li Y.Z. (2011). Rapid determination of volatile constituents in safflower from Xinjiang and Henan by ultrasonic-assisted solvent extraction and GC-MS. J. Pharm. Anal..

[7] Zhang H.L., Nagatsu A., Sakakibara J. (1996). Novel antioxidants from safflower (*Carthamus tinctorius* L.) oil cake. Chem. Pharm. Bull..

[8] Zhang H.L., Nagatsu A., Watanabe T., Okuyama H. (1997). Antioxidative compounds isolated from safflower (*Carthamus tinctorius* L.) oil cake. Chem. Pharm. Bull..

[9] Shebis Y., Iluz D., Kinel-Tahan Y., Dubinsky Z., Yehoshua Y. (2013). Natural antioxidants: Function and sources. Food Nutr. Sci..

[10] Wakayama S., Kusaka K., Kanehira T., Yamada Y., Kawazu K., Kobayashi A. (1994). Kinobeon A, a novel red pigment produced in safflower tissue culture systems. Z. Nat. C.

[11] Erichsen-Brown C. (2013). Medicinal and Other Uses of North American Plants: A Historical Survey with Special Reference to the Eastern Indian Tribes.

[12] Nascimento G.G., Locatelli J., Freitas P.C., Silva G.L. (2000). Antibacterial activity of plant extracts and phytochemicals on antibiotic-resistant bacteria. Braz. J. Microbiol..

[13] Saxena G., McCutcheon A., Farmer S., Towers G., Hancock R. (1994). Antimicrobial constituents of *Rhus glabra*. J. Ethnopharmacol..

[14] Deve A.S., Kumaresan K., Rapheal V.S. (2014). Extraction process optimization of polyphenols from Indian *Citrus sinensis* as novel antiglycative agents in the management of diabetes mellitus. J. Diabetes Metab. Disord..

[15] Sharma R., Tahiliani S., Jain N., Priyadarshi R., Chhangani S., Purohit S., Joshi P. (2013). Cynodon dactylon leaf extract assisted green synthesis of silver nanoparticles and their anti-microbial activity. Adv. Sci. Eng. Med..

[16] El-Shahaby O., El-Zayat M., Salih E., El-Sherbiny I., Reicha F. (2013). Evaluation of antimicrobial activity of water infusion plant-mediated silver nanoparticles. J. Nanomed. Nanotechnol..

[17] Yadav R.N.S., Agarwala M. (2011). Phytochemical analysis of some medicinal plants. J. Phytol..

[18] Li J.-E., Fan S.-T., Qiu Z.-H., Li C., Nie S.-P. (2015). Total flavonoids content, antioxidant and antimicrobial activities of extracts from *Mosla chinensis* Maxim. cv. Jiangxiangru. LWT Food Sci. Technol..

[19] Christodouleas D.C., Fotakis C., Nikokavoura A., Papadopoulos K., Calokerinos A.C. (2015). Modified DPPH and ABTS assays to assess the antioxidant profile of untreated oils. Food Anal. Methods.

[20] Gaidhani S., Singh A., Kumari S., Lavekar G., Juvekar A., Sen S., Padhi M. (2013). Evaluation of some plant extracts for standardization and anticancer activity. Indian J. Tradit. Knowl..

[21] CoStat (2017). Statistical Software Version 6.450: CoHort Software, 798 Light houseAve, PMB 320.

[22] Duncan D.B. (1955). Multiple range and multiple F tests. Biometrics.

[23] Aliakbarlu J., Mohammadi S., Khalili S. (2014). A study on antioxidant potency and antibacterial activity of water extracts of some spices widely consumed in Iranian diet. J. Food Biochem..

[24] Egorova E., Revina A. (2000). Synthesis of metallic nanoparticles in reverse micelles in the presence of quercetin. Colloids Surf. A Physicochem. Eng. Asp..

[25] Ghosh S., Patil S., Ahire M., Kitture R., Kale S., Pardesi K., Cameotra S.S., Bellare J., Dhavale D.D., Jabgunde A. (2012). Synthesis of silver nanoparticles using *Dioscorea bulbifera* tuber extract and evaluation of its synergistic potential in combination with antimicrobial agents. Int. J. Nanomed..

[26] Abu-Reidah I.M., Ali-Shtayeh M.S., Jamous R.M., Arráez-Román D., Segura-Carretero A. (2015). HPLC–DAD–ESI-MS/MS screening of bioactive components from *Rhus coriaria* L.(Sumac) fruits. Food Chem..

[27] Sroka Z., Cisowski W. (2003). Hydrogen peroxide scavenging, antioxidant and anti-radical activity of some phenolic acids. Food Chem. Toxicol..

[28] Harper A., Kerr D.J., Gescher A., Chipman J.K. (1999). Antioxidant effects of isoflavonoids and lignans, and protection against DNA oxidation. Free Radic. Res..

[29] Ng T., Liu F., Wang Z. (2000). Antioxidative activity of natural products from plants. Life Sci..

[30] Lobo V., Patil A., Phatak A., Chandra N. (2010). Free radicals, antioxidants and functional foods: Impact on human health. Pharmacogn. Rev..

[31] Birben E., Sahiner U.M., Sackesen C., Erzurum S., Kalayci O. (2012). Oxidative stress and antioxidant defense. World Allergy Organ. J..

[32] Butovich I.A., Lukyanova S.M. (2008). Inhibition of lipoxygenases and cyclooxygenases by linoleyl hydroxamic acid: Comparative in vitro studies. J. Lipid Res..

[33] Viji V., Helen A. (2008). Inhibition of lipoxygenases and cyclooxygenase-2 enzymes by extracts isolated from *Bacopa monniera* (L.) Wettst. J. Ethnopharmacol..

